# Central Dental Association of Northern New Jersey

**Published:** 1888-05

**Authors:** 


### CENTRAL DENTAL ASSOCIATION OF NORTHERN NEW JERSEY.

### REGULAR MEETING FOR OCTOBER, 1887.

Reported for the Independent Practitioner.

A paper was read by Dr. E. S. Niles, of Boston, upon "Dead and Diseased Teeth and their Treatment," (see page 230). During the reading of the paper the author referred to charts illustrating the subject, and he explained them as follows:—

The pulp having died, the infective matter has found its way out into the tissues, and they have become inflamed and irritated. Suppuration ensues, gases are formed, and finally the pus burrows to the surface here (referring to drawing). This is a condition that I understand is called alveolar abscess. In a case of this kind the diseased condition may go on to that extent that the alveolus itself becomes diseased, dead bone is formed, and it is necessary to cut it away.

I have never seen but one case in which the extraction of the tooth did not eradicate the disease, and I have come to the conclusion that although the alveolus is affected, the tooth is the cause of the trouble. If the extraction of the tooth cures the disease, it evidently comes from the tooth; and if the tooth is hermetically sealed the tissues will tolerate it, and it will become a useful organ.

If we allow arsenic to get to the end of the root it will always cause trouble. If I get to the end of a tooth in which the pulp is alive with my broach, when I withdraw it there is usually no inflammation following. The pulp heals over when no arsenic has reached that point. I cannot see any of the root, yet theoretically that is the method that should be pursued. I know that a great many are inclined to make a free opening. I always fear an opening of that kind, where wounded tissue will be left and a condition be set up that would cause inflammation.

Merely wiping the canal with cotton is hardly sufficient to dry it into the dentine to any extent. It is often forgotten or overlooked that the dentine itself takes up the infectious matter, and that very often this matter may get into the canal, and this would not take place if it had been dried. I always dry as much as possible, both in filling and in antiseptic treatment.

*Dr. Atkinson*—I am apt to characterize things by the names that best suit them. I was never more astonished in my life than at the presentment that has been made to-night. If the essayist has really given us a synopsis of what he has done, or if this is his experience, he needs our sympathies. I should like to know what he means by a twenty per cent. solution and a five per cent. solution of bichloride of mercury.

*Dr. Niles*—A twenty per cent. solution is one consisting of twenty parts of bichloride of mercury and eighty parts of water, and a five per cent. solution consists of five parts of the bichloride and ninety-five parts water.

*Dr. Atkinson*—It will not dissolve at an ordinary temperature, or without some adventitious aid, and it leaves a precipitate, showing that the whole is not held in solution.

What becomes of pulps that are senile ? The senile condition is a minified blood circulation and an increased calcareous deposit, that has many modes of expression. All those who have extracted teeth of old persons will remember that the roots are nearly as transparent as glass, showing that the red blood no longer circulates in the remnants of the pulp-chamber that is always found on the side of the deposit of secondary dentine. The metamorphosis that occurs is toward the embryonic condition, and it is not such that the pulp cannot die till there is a microbe there.

Let us follow this pulp business. The chances are that in the cases cited there was an excessive quantity of lime-salts present, and if you have the ability to drill through and let out the pus you will be able to restore the tooth, by proper treatment, to a healthy condition without loss of the pulp. I have found teeth that did well in that condition. The change that takes place in an exposed pulp is usually partial or complete atrophy, of which there are several kinds. There may be simple recession of the cornua of the pulp, leaving the chamber dry and the balance of the bulbous portion of the pulp living; or atrophy may extend throughout the entire bulbous portion of the pulp, leaving the chamber full of a gelatinous mass of hyper-oxidized hydrate of carbon, with normal pulps in the roots; or it may die to the ends of the roots and be converted into a hyaline or jelly-like mass, where oxy-chloride has been used; or it may die by a conversion into an atheromatous condition, known as fatty degeneration, akin to what is seen in corpses under the name of adipocere. All these changes are odorless and benign. But where the return to the embryonic condition is so rapid as to initiate the metamorphosis of the tissue so rapidly as to disintegrate the embryonal corpuscles into pus corpuscles and set free ammoniacal and carburetted gases, the pulp is offensive to smell and needs extirpation; then after disinfecting such a canal, seal up and restore the contour of the tooth. If the gentleman can take out the fibrils in the tubuli of the canal, and disinfect the little canaliculi, I will thank him to instruct me how to do it. I have never known an instance where a fibril has been taken out in such a condition. Dr. Barrett said he had seen a solution of gutta-percha in chloroform ooze out from the tubules into the pericementum.

I have put four grains of bichloride of mercury into an ounce of water, and have always found a precipitate that was not dissolved, showing what was supposed to be the first point of saturation at an ordinary temperature. I have seen hydro-chloro-zincate of albumen filling the entire pulp-chamber of a cuspid to the end of the root, and no evidence of disease whatever. I extirpated this, and dressed it for a few days before filling. I would not do that again. I would immediately fill. I think nature knows a little of what she is about in these cases, and we should do just as she does; either take out the teeth, excommunicate or encyst them. All things that are encysted are absolutely excommunicated.

When you have taken out a pulp without going through the foramen entirely, there is where you get into trouble. Go through the foramen, and then wash out and treat it with water about as salt as sea water, or with the bichloride of mercury, or any of those agents that hold oxygen with such a loose grip, and you will be astonished to see how nicely it will get along. When a pulp is wholly dead it will be dead all the way around and through the canal.

*Dr. Luckey*—I cannot endorse all the methods that the gentleman has recommended. I think that in cases of dead pulps some antiseptic treatment is necessary, and that the practice of immediate root filling where there has been inflammation and abscess is injudicious. The proper treatment, in my opinion, is to clean out the canal and disinfect the whole thing, the abscess sac and also the canaliculi, and close up the external opening.

*Dr. Atkinson*—What is better than salt water for cleansing the canal ?

*Dr. Luckey*—I think listerine is better.

*Dr. Atkinson*—It is useful as being a loose holder of oxygen. In the case of Daniel S. Dickerman there was half of a left superior central that was still good, the other half having broken down. There was a fistulous abscess that had continued for twenty-four years. It was cleaned out to the lingual border, bleached and filled, and in four weeks was sound, and has remained so.

*Dr. Luckey*—There are exceptions to all rules. One thing Dr. Atkinson taught me years ago, and that was to go through the end of the root with the drill, and if the experience of other gentlemen has been the same as mine, they have found it not to be safe practice. Our Swiss broaches are usually sufficient to get there. If there is an abscess, the probabilities are that the canal will be enlarged sufficiently to permit medicines to pass through, and if it is so enlarged it is a very difficult matter to fill it. I think the gentlemen present will bear me out in saying that there are a greater number of failures than successes in immediate root filling.

*Dr. Stockton*—This does indeed seem to be a live subject. The

Chicago Dental Society issued a pamphlet on the subject, and it was more fully discussed and excited more attention, both at Niagara Falls and Old Point Comfort, than any other ; you remember that at our own State Society Meeting the same interest was manifested in the subject, and now again to-night, by way of Boston, it comes to us. In all these discussions of the last season I have been unable to reach a sure, definite mode of treating pulpless teeth.

I believe in removing pulps if they are exposed and have given pain, and I believe in doing so without the aid of arsenic. Open well the pulp-chamber, apply cocaine for a few minutes and then "knock it out." The pain is not very severe, and is very, very much preferable to the pain incident to the arsenic. The knocking-out is but for an instant, while the pain from the arsenic continues for hours, and even then the final removal is oftentimes as painful as the knocking-out. Then too, when the knocking-out process is used, the pulp-chamber and crown can be filled at once, and no after trouble is likely to ensue. In all cases, when there is a fistulous opening, cleanse thoroughly and fill at once, and the opening will heal and the tooth become healthy, firm and useful.

In cases of blind abscess, I cleanse thoroughly, using a solution of bichloride of mercury 1 part to about 500 water for syringing the canal, and when satisfied that I have cleaned out all the dead matter I use the peroxide of hydrogen, and do so until there is no evidence of pus. Then after thoroughly drying the canal, I fill it with gutta-percha dissolved in chloroform, in which I have incorporated hydronaphthol, and if every step has been thoroughly done you can rest assured of very little future trouble.

The simple cleaning out of the putrescent pulp will in many cases bring about a cure. The tooth is so delighted to be clean that it at once gets well. If trouble should follow filling, then treat thorough the alveolus. But fill the roots. Many, very many teeth come to me abscessed because the dead pulp has never been removed and the root filled.

*Dr. Osmun*—Did I understand Dr. Niles to say that all fistulous teeth belong to the fourth class ?

*Dr. Niles*—That might be.

*Dr. Osmun*—That is a point that I wish to get clear to my mind. I have never met with any such teeth that could be denominated as belonging to the fourth class. I have had to treat some. I want to raise my voice against long treatment. I also want to protest against immediate root filling. It requires some practice to know when to fill and when to wait. My own experience has led me to the conclusion that in the treatment of pulpless teeth it would be absolutely impossible to lay down any infallible rule. It has impressed itself upon my mind that after three or four treatments, if they have been thorough, you can safely leave it to the care of nature without fear of any further trouble. I believe the days of capping pulps have well nigh gone by. There are pulps that can be capped successfully, and if there are ever so many failures it is no reason why all should be failures. I have made such operations that have been successful, and I have many others where the pulp has gone the way of all flesh. I am glad that Dr. Niles has read this paper, for I believe there is no subject that opens a broader field for discussion than this one of dead pulps.

*Dr. Palmer*—I think Dr. Osmun and Dr. Luckey have given the impression that it would be wrong to fill at the first sitting.

*Dr. Luckey*—Yes.

*Dr. Palmer*—I think Dr. Luckey would not object to filling immediately in cases where the pulp has just been extirpated. I should do so in such cases. Dr. Luckey speaks of the canals or tubuli being filled with matter. Why cannot you disinfect them ?

*Dr. Luckey*—Because you cannot force your medicine into the openings.

*Dr. Odell*—If it were a tooth in which you had extirpated the pulp, possibly you might have trouble. If you have nothing in this canal but a decomposed material, I do not see why you cannot fill it.

*Dr. Luckey*—For this reason; that the little tubules are filled with mephitic gases. You might disinfect the pulp-chamber.

*Dr. Atkinson*—It is simply a question of the arrest of the formation of gases, and you will always do that when you arrest the inflammation and seal up the canal.

*Dr. Luckey*—What becomes of the gases that are confined in the tubules ?

*Dr. Atkinson*—They are so confined that they cannot kick. Put a pressure here, and as long as there is a tension from the incoming oxygen the fermentation will continue.

*Dr. Luckey*—Some of these fibers come in from the pulp-canal, others from the periphery of the tooth, and others from the pericementum.

*Dr. Atkinson*—They go crooked.

*Dr. Palmer*—There is a certain amount of life surrounding a tooth which it receives independent of the pulp, I admit.

*Dr. Atkinson*—When a tooth is not loose or tender, fill it.

*Dr. Palmer*—It seems to me that if these tubuli were filled with mephitic gases, the cleansing solution should be strong enough to disinfect them, although I am not enough of a chemist to explain the proportions. In cases where the pulp has recently been extirpated there should be no hesitancy in regard to immediate filling, and where there is a fistulous opening in the gum, it seems to me good practice to cleanse, disinfect and thoroughly fill at once, for nature will heal the fistula. I did so on Saturday in a case where the opening had existed for at least four years, and to-day the process of repair is going on nicely. I doubt very much the accuracy of Dr. Luckey's statement that there are more failures than successes following this treatment. I use iodoform usually, sometimes with, sometimes without, carbolic acid.

*Dr. J. Bond Littig*—Mr. President and gentlemen, I came in so late that I did not hear all of Dr. Niles' paper. From the discussion that I have heard I think that there is a good deal of hair-splitting. In cases of fistulous opening I should simply pump through the foramen carbolic acid and disinfect the tooth, and then fill it. I have seldom any trouble with a tooth so treated. I would treat so long as there is any odor. I usually fill with silk, which I withdraw at the next sitting, and if there is no discoloration of the silk I proceed to fill. I think I can say that I save ninety per cent. of the teeth so filled. I do not think so much of gutta-percha filling as some do, for I find that when I take out the gutta-percha it has a bad odor. I prefer chloride of zinc. I think it preserves the canal better and keeps it in a more aseptic condition. That is the treatment I have been pursuing for a great number of years. I do not think there is so much difficulty about it after all; the teeth usually get along, but sometimes I have trouble, owing to the constitutional condition of the patient. There is only one class of cases in which the teeth have caused me any trouble, and that is teeth that have been broken by an accident, in which case I use the treatment of Dr. Howe, and fill with iodoform.

Not having heard all of the paper I cannot speak of it very intelligently. There is one point on which I cannot agree with Dr. Niles. I do not apply arsenic to aching teeth.

*Dr. Niles*—In going into the details of my treatment of the cases which have come into my hands, in no sense did I wish to imply that the same treatment was not used by the dentists of New Jersey. I was specially assisted by Dr. Herman, of South Boston, in the method of treating canals by the use of watch-spring broaches. They are much better, I find, than those which are generally used by dentists. There are several points that Dr. Atkinson has brought up. One as to the bichloride of mercury. He says the bichloride of mercury as now prepared cannot be dissolved in water. He seems to have made the mistake of taking calomel for bichloride of mercury. He has spoken of alcohol; alcohol will not dissolve albumen. As regards salt, it has no antiseptic power at all; it is simply a cleanser.

